# Design of Locally Resonant Acoustic Metamaterials with Specified Band Gaps Using Multi-Material Topology Optimization

**DOI:** 10.3390/ma17143591

**Published:** 2024-07-20

**Authors:** Hongfang Chen, Yu Fu, Ling Ling, Yujin Hu, Li Li

**Affiliations:** State Key Lab of Intelligent Manufacturing Equipment and Technology, School of Mechanical Science and Engineering, Huazhong University of Science and Technology, Wuhan 430074, China; hongchf@hust.edu.cn (H.C.); fuyu_mse@hust.edu.cn (Y.F.); lili_em@hust.edu.cn (L.L.)

**Keywords:** locally resonant acoustic metamaterials, band gaps with specified frequency, homogenization framework, multiple materials, parameterized level-set method

## Abstract

Locally Resonant Acoustic Metamaterials (LRAMs) have significant application potential because they can form subwavelength band gaps. However, most current research does not involve obtaining LRAMs with specified band gaps, even though such LRAMs are significant for practical applications. To address this, we propose a parameterized level-set-based topology optimization method that can use multiple materials to design LRAMs that meet specified frequency constraints. In this method, a simplified band-gap calculation approach based on the homogenization framework is introduced, establishing a restricted subsystem and an unrestricted subsystem to determine band gaps without relying on the Brillouin zone. These subsystems are specifically tailored to model the phenomena involved in band gaps in LRAMs, facilitating the opening of band gaps during optimization. In the multi-material representation model used in this method, each material, except for the matrix material, is depicted using a similar combinatorial formulation of level-set functions. This model reduces direct conversion between materials other than the matrix material, thereby enhancing the band-gap optimization of LRAMs. Two problems are investigated to test the method’s ability to use multiple materials to solve band-gap optimization problems with specified frequency constraints. The first involves maximizing the band-gap width while ensuring it encompasses a specified frequency range, and the second focuses on obtaining light LRAMs with a specified band gap. LRAMs with specified band gaps obtained in three-material or four-material numerical examples demonstrate the effectiveness of the proposed method. The method shows great promise for designing metamaterials to attenuate specified frequency spectra as required, such as mechanical vibrations or environmental noise.

## 1. Introduction

LRAMs have some interesting phenomena, one of which is that they can form subwavelength band gaps. This gives LRAMs significant potential for applications in isolating low-frequency vibrations or noise using a small-sized structure. Liu et al.’s work demonstrated that composites consisting of LRAMs exhibit subwavelength band gaps [1]. Later, an analytical model was introduced where effective mass densities might become negative near local resonances [2]. Acoustic metamaterials, with their inherent deep subwavelength nature, have triggered many exciting investigations, as reviewed in [3,4].

In this work, a topology optimization method is used to obtain multi-material LRAMs with the objective of isolating specified frequency ranges of vibration or noise. Using this proposed method, LRAM band gaps can be designed as required within a frequency range. The frequency limitations when the unit cell size and materials are specified can be found in Roca et al.’s work [5]. Unlike traditional methods, topology optimization methods can provide innovative structural configurations under different objectives and constraints [6]. Among these topology optimization methods, solid isotropic material with penalization (SIMP) methods [7,8,9,10], evolutionary structural optimization (ESO) methods [11,12], and level-set methods (LSMs) [13,14] are commonly used in many fields.

As reviewed in Li et al.’s paper [15], since topology optimization techniques were introduced in acoustic metamaterial designs, many innovative works have been achieved. The target of this paper is to study the band-gap optimization problems of LRAMs, a topic that has attracted great interest from researchers. In 2003, Sigmund et al. utilized a topology optimization technique for the first time to obtain wider band gaps in phononic crystals [16]. T. Matsuki et al. [17] used the optimization technique to design LRAMs. Yang et al. [18] maximized the first band gap of LRAMs using the concept of effective mass density. Roca et al. [5] introduced an approach to maximize LRAM band gaps, where a multiscale homogenization framework combined with model order-reduction techniques was introduced to build the optimization model. Zhang et al. [19] designed band gaps in acoustic metamaterials based on a material-field series expansion framework. To obtain low-frequency broad band gaps, Sun et al. [20] improved the scatterer filling scheme in two hierarchical honeycomb metamaterials. Li et al. [21] sought anisotropic hierarchical honeycomb acoustic metamaterials with multiple broad band gaps using a topology optimization method.

Since an LRAM typically consists of a matrix, coating, and scatterer, the material parameters of these components can be significantly different [1,2,18]. For instance, the stiffness of the coating is lower by several orders of magnitude compared to that of the scatterer. This makes it difficult to use a single material to obtain LRAMs with band gaps relying on the local resonance mechanism, whereas the use of multiple materials can make this easier. This paper introduces a method that can utilize multiple materials to obtain LRAMs with band gaps that satisfy specified frequency constraints. Clear boundaries among the individual constituent materials are beneficial for LRAM band-gap optimization with multiple materials. Therefore, this work utilizes a parameterized level-set method (PLSM). Almost two decades ago, the LSM was introduced to address structural optimization problems [22,23]. Later, by modifying the original method, the PLSM was presented for shape and topology optimization [24,25]. Wang and Wang [26] presented a model for multi-material problems, in which $n=2^{m}$ material phases are denoted by *m* level-set functions. Wang et al. [27] introduced a model for multi-material problems, where a combined formulation of level-set functions is used to represent each phase. Using multiple materials, Vogiatzis et al. [28] adopted a reconciled LSM to address the optimization problems of negative Poisson’s ratio metamaterials. Liu and Ma [29] introduced another model that employed *p* variables to denote *p* materials along with the void. Xia and Shi [30] employed an ESO technique to tackle hole nucleation problems in an LSM for multi-material optimizations. Using the alternating active-phase algorithm and the LSM, Sha et al. [31] effectively addressed optimization problems involving multiple materials. Besides LSMs, there are certainly other types of multi-material approaches. Bendsøe and Sigmund [32] proposed a strategy using the SIMP method to interpolate two materials and a void phase. Hvejsel and Lung [33] expanded on the aforementioned SIMP method by introducing a generalized interpolation scheme that can be applied to any number of materials. Gao and Zhang [34] discussed density-based multi-material interpolation techniques through numerical tests and theoretical analysis. Tavakoli and Mohseni [35] suggested an alternating active-phase algorithm, where the problem was partitioned as a series of two-phase sub-problems. Zuo and Saitou [36] presented the ordered SIMP approach, aimed at improving computational efficiency. Huang and Xie [37] introduced an ESO method for optimization problems involving both single and multiple materials.

Despite considerable progress, the topology optimization method for LRAMs is still not mature. Most current research does not involve obtaining LRAMs with specified band gaps. However, such LRAMs have significant application potential. This is because LRAMs can form subwavelength band gaps, which enable them to isolate vibrations or noise in specified low-frequency ranges. Utilizing multiple materials in LRAM optimization may help achieve results with a sufficiently wide band gap while simultaneously meeting other objectives, such as mass reduction. Therefore, this work proposes a multi-material topology optimization approach for designing LRAMs with specified band gaps. The band-gap calculation in this method uses a homogenization framework [38,39], which establishes both a restricted subsystem and an unrestricted subsystem to determine the band gaps. These subsystems are specifically tailored for LRAMs, facilitating the opening of band gaps based on the local resonance mechanism during optimization. Sensitivity analysis only needs to consider these two subsystems. Consequently, sensitivity information related to band gaps can be calculated without relying on the Brillouin zone. This implies that the proposed method simplifies the optimization calculation. In the representation model of this multi-material optimization method, each material, except for the matrix material, is represented by a similar combinatorial formulation of level-set functions. This model reduces the direct conversion between materials other than the matrix material, thereby enhancing the band-gap optimization of LRAMs. The effectiveness of this method is tested by applying it to solve two types of LRAM optimization problems. The first test involves maximizing the band-gap width while ensuring it encompasses a specified frequency range, and the second test focuses on obtaining light LRAMs with a specified band gap. Numerical examples successfully yield LRAM designs with the required band gaps using three or four materials, demonstrating the feasibility and effectiveness of this approach. In our future work, we will manufacture the obtained LRAMs and analyze their properties experimentally, similar to studies of other types of materials [40,41].

This paper is structured as follows. Section 2 introduces a simplified calculation method for LRAM band gaps. Section 3 proposes the mathematical model for multi-material LRAMs. Section 4 utilizes numerical examples to prove the feasibility of the presented method. The conclusions are provided in Section 5.

## 2. A Simplified Calculation Method for LRAM Band Gaps Based on a Homogenization Framework

**Figure 1:**
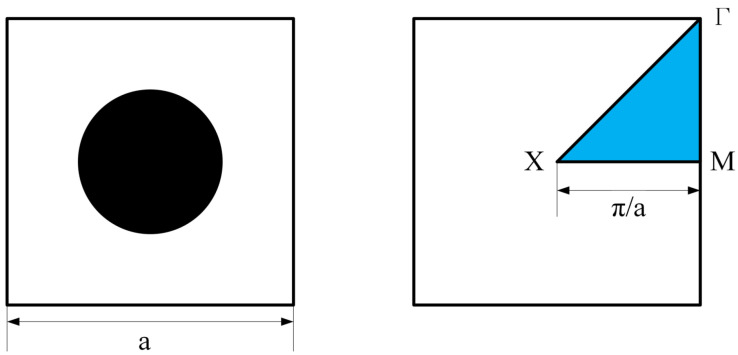
The square unit cell and its corresponding irreducible Brillouin zone (blue region).

Generally, the elastic wave field in LRAMs can be represented as a periodic function. Following the Bloch theorem, band-gap analysis can be simplified to consider only a single LRAM cell [42,43,44,45,46]. The square unit cell under study and its corresponding irreducible Brillioun zone are presented in Figure 1. According to existing research [16,47,48], the computation of band diagrams is reduced by only considering the boundary of the irreducible Brillouin zone. Therefore, in conventional optimization methods, calculating the band gap of LRAMs typically involves traversing the Γ-X-M-Γ path of the Brillouin zone. The computational cost is significantly impacted by the number of selected wave vectors [16,49,50]. This paper introduces a simplified calculation method for LRAM band gaps based on a homogenization framework. The eigenfrequencies obtained from two subsystems are used to determine the LRAM band gaps.

### 2.1. Dispersion Curve Analysis of LRAMs

Generally, one can identify whether there are band gaps through dispersion curve analysis of LRAMs. The propagation characteristics of elastic waves in isotropic elastic materials have been extensively researched. In this paper, the analysis method used in Li et al.’s work [49] is adopted. The governing equation is

(1) $$▿\left[\lambda \left(\gamma \right)+2\mu \left(\gamma \right)\right]\left(▿\cdot u\right)-▿\times \left[\mu \left(\gamma \right)▿\times u\right]=\rho \left(\gamma \right)\ddot{u},$$

where γ is the location vector, λ and μ represent the Lame’s coefficients, u denotes the displacement vector, and ρ is the mass density. For two-dimensional LRAMs, the elastic wave propagation mode includes two coupled in-plane modes and one out-of-plane mode. In this paper, only in-plane waves are considered.

In an infinite periodic structure, according to Bloch’s theorem, the displacement vector $u\left(\gamma ,k\right)$ is expressed as

(2) $$u\left(\gamma ,k\right)=u_{k}\left(\gamma \right)e^{i(k\cdot \gamma )},$$

where $u_{k}\left(\gamma \right)$ is a periodic function of the amplitude displacement and $k=\left(k_{x},k_{y}\right)$ represents the wave vector.

By substituting Equation (2) into the governing equations for in-plane waves, one obtains a generalized eigenvalue equation that can be solved using finite-element methods [51,52]:

(3) $$Kv=\omega^{2}Mv,$$

where K and M are the stiffness and mass matrices, respectively; v denotes the eigenvectors; and ω represents the angular frequency.

### 2.2. The Establishment of Subsystems Based on the Homogenization Framework

The homogenization framework utilizing the Craig–Bampton reduction technique [38] and the framework based on the extended Hill–Mandel principle [39] is tailored for modeling the phenomena involved in LRAMs. Both frameworks can be used to calculate LRAM band gaps. When applying the framework presented by Roca et al. [39] in practice, boundary conditions need to be considered [5]. Therefore, by incorporating techniques from this framework with the Craig–Bampton method [53], the band-gap calculation method can be derived in a simple and direct way. The method presented in Roca et al.’s work [39] is used to establish the relationship between the microstructure and the macroscale and to capture the local resonance phenomenon. The Craig–Bampton technique is employed to generate compact, reduced models of microstructures, aiding in the computation of local resonance effects. Based on these two works, an unrestricted subsystem and a restricted subsystem are established to help calculate the band gaps of LRAMs. This method incorporates the characteristics of the two previously mentioned homogenization frameworks and can be easily implemented using finite-element methods.

In the homogenization scheme, to maintain the separation of scales, it is essential to ensure that the microstructure size is far smaller than the wavelength. This work is based on two main hypotheses: (1) kinematic admissibility between scales; and (2) the principle of virtual work, which states that the total internal virtual work remains constant for a dynamic system regardless of the imposed kinematics. Through homogenization, the solution to the microscale problem can be used to derive the macroscale constitutive response. Since this paper focuses on establishing the optimization computational model, more detailed information about the theoretical aspects can be found in [38,39].

The selected representative volume element (RVE) is the unit cell of the LRAM. This paper only studies two-dimensional problems without considering plane rotation. The response of the RVE to the applied force can be evaluated by solving

(4) $$M\ddot{u}+\mathrm{Ku}=F,$$

where M represents the mass matrix, K denotes the stiffness matrix, u and $\ddot{u}$ represent the displacement and the acceleration, and F stands for the applied force.

**Figure 2:**
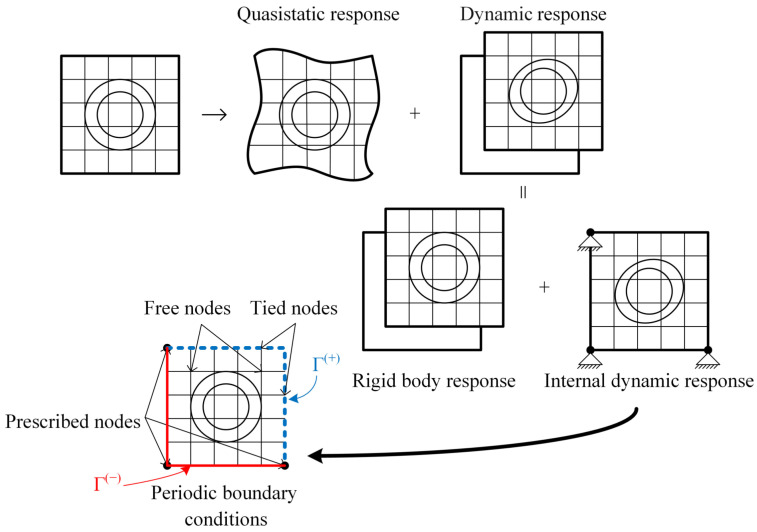
Illustration of the total response of the structure and the representation of the periodic boundaries in a typical 2D square unit cell. Based on this, the subsystems are established.

The responses can be split into two systems using the decomposition method. One is a quasistatic system, and the other is an internal dynamic system. The overall structural response can be represented as the sum of its quasistatic response and its internal dynamic response. The internal dynamic system can be built as an unrestricted subsystem or a restricted subsystem, through which one can determine the LRAM band gap [39]. The contribution of the restricted subsystem to the macroscale consists of the rigid body displacement and the internal dynamic response. The internal dynamic response can be projected onto the space spanned by the eigenmodes of the microstructure, with the prescribed nodes fixed. This entire process is illustrated in Figure 2. The unrestricted subsystem is a generalized eigenvalue problem, whose contribution to the macroscale is discussed in Section 2.3.2.

Use β to represent the local macroscale homogenized force

(5) $$\beta =I^{T}F,$$

where

(6) $$I=\left[\begin{array}{c} \;\;\;\vdots \\ 1\;0\\ 0\;1\\ \;\;\;\vdots \end{array}\right].$$

For simplicity, the typical two-dimensional square unit cell is considered, as depicted in Figure 2. The system is divided into tied nodes and retained nodes to facilitate the application of periodic boundary conditions. The tied nodes consist of the right and top edges and the upper-right vertex. In the calculation, the retained nodes are subdivided into prescribed nodes and free nodes to facilitate problem-solving [38]. Since the responses of tied nodes can be obtained through other nodes based on periodic boundary conditions, the LRAM cell can be partitioned into prescribed nodes, denoted by ‘1’, and remaining nodes, denoted by ‘2’, to derive the response

(7) $$\begin{array}{c} \left[\begin{array}{c} M_{11}\;M_{12}\\ M_{21}\;M_{22} \end{array}\right]\left[\begin{array}{c} {\ddot{U}}_{\mu 1}\\ {\ddot{U}}_{\mu 2} \end{array}\right]+\left[\begin{array}{c} K_{11}\;K_{12}\\ K_{21}\;K_{22} \end{array}\right]\left[\begin{array}{c} U_{\mu 1}\\ U_{\mu 2} \end{array}\right]=\left[\begin{array}{c} F_{1}\\ 0 \end{array}\right], \end{array}$$

where $U_{\mu }$ and ${\ddot{U}}_{\mu }$ represent the displacement and acceleration associated with each node.

In calculating the quasistatic response, the mass contribution of the system is omitted. The following relationship is derived using the condensation method:

(8) $$K_{21}U_{\mu 1}+K_{22}U_{\mu 2}=0.$$

The target of this paper is to study LRAM band-gap optimization problems, so we do not pay much attention to the quasistatic response.

In the restricted dynamic subsystem, the prescribed nodes do not participate in the internal vibrations. Based on the second equation of system (7), one can obtain

(9) $$M_{22}{\ddot{U}}_{\mu 2}+K_{22}U_{\mu 2}+M_{21}{\ddot{U}}_{\mu 1}+K_{21}U_{\mu 1}=0.$$

Use $\hat{\left(\cdot \right)}$ to indicate that the periodic boundary condition is imposed; thus,

(10) $${\hat{M}}_{22}{\ddot{\hat{U}}}_{\mu 2}+{\hat{K}}_{22}{\hat{U}}_{\mu 2}=-\left({\hat{M}}_{21}{\ddot{\hat{U}}}_{\mu 1}+{\hat{K}}_{21}{\hat{U}}_{\mu 1}\right).$$

Through Equation (10), the following subsystem can be obtained:

(11) $$M_{\mu 2}^{*}{\ddot{\hat{U}}}_{\mu 2}^{*}+K_{\mu 2}^{*}{\hat{U}}_{\mu 2}^{*}=-D\ddot{u},$$

where $M_{\mu 2}^{*}$ and $K_{\mu 2}^{*}$ represent the mass and stiffness matrices obtained after imposing the restrictions, respectively. ${\hat{U}}_{\mu 2}^{*}$ is the fluctuation field vector, and D represents a matrix coupling the accelerations of the micro- and macroscales.

The macroscale inertial force derived is as follows:

(12) $$\beta =\bar{R}\ddot{u}+D{\ddot{\hat{U}}}_{\mu 2}^{*}.$$

Derivation details for Equations (11) and (12) are given in Appendix A.

### 2.3. A Simplified Calculation Method for LRAM Band Gaps Using Subsystems

In this section, the contribution of the microfluctuation field to the macroscale is analyzed. Subsequently, a simplified calculation method for the band gap of LRAMs is established.

In this paper, wave solutions are considered. The vector of nodal values ${\hat{U}}_{\mu }$ is

(13) $${\hat{U}}_{\mu }=Ue^{i(\kappa n_{\kappa }\cdot x-\omega t)},$$

where U represents the amplitude function of ${\hat{U}}_{\mu }$, x denotes the spatial coordinate, ω represents the angular frequency, $n_{\kappa }$ signifies the wave propagation direction, and κ stands for the matching wavenumber.

#### 2.3.1. The Map of the Restricted Subsystem to the Macroscale

This subsection analyzes the contribution of the microfluctuation field from the restricted subsystem to the macroscale. For the interior free vibration system, the eigenvalue problem is given as

(14) $$(K_{\mu 2}^{*}-\omega_{\mu }^{*2}M_{\mu 2}^{*}){\hat{\psi }}_{\mu 2}^{*}=0,$$

where ${\hat{\psi }}_{\mu 2}^{*}$ is the natural vibration mode and $\omega_{\mu }^{*}$ is the corresponding eigenfrequency. Solving the generalized eigenvalue problem of interior dynamics yields eigenmodes. These eigenmodes are then used as a reduced basis in subsequent calculations to capture the local resonance phenomenon and derive the macroscale inertial force

(15) $$\beta =\bar{R}\ddot{u}+Q{\left(\Omega_{\mu }^{*2}/\omega^{2}-I\right)}^{-1}Q^{T}\ddot{u},$$

where $\bar{R}$ represents the effective density tensor, the derivation of which can be found in Appendix A. Q is a coupling matrix. The symbol $\Omega_{\mu }^{*}$ represents a diagonal matrix that holds the natural frequencies $\omega_{\mu }^{*}$ of the restricted subsystem. The derivation process is given in Appendix B.1.

Using $R^{e}$ to represent the effective pseudo-density tensor, one can obtain

(16) $$R^{e}=\bar{R}+\tilde{R},$$

where

(17) $$\tilde{R}=Q{\left(\Omega_{\mu }^{*2}/\omega^{2}-I\right)}^{-1}Q^{T}.$$

#### 2.3.2. The Map of the Unrestricted Subsystem to the Macroscale

Consider the generalized eigenvalue problem formed by the unrestricted subsystem, where the design domain is not partitioned into prescribed and retained nodes

(18) $${\hat{M}}_{\mu }{\ddot{\hat{U}}}_{\mu }+{\hat{K}}_{\mu }{\hat{U}}_{\mu }=N_{\mu }^{T}\beta ,$$

where $N_{\mu }$ represents the shape function. $\hat{\left(\cdot \right)}$ denotes that the periodic boundary condition has been imposed.

The macroscale inertial force β can be obtained through the generalized eigenvalue problem of the unrestricted subsystem

(19) $$\Psi_{\mu }{\Psi_{\mu }}^{T}N_{\mu }^{T}\beta =(\Omega_{\mu }^{2}-\omega^{2})Ue^{i(\kappa n_{\kappa }\cdot x-\omega t)},$$

where

(20) $$\left\{\begin{array}{c} \Psi_{\mu }=\left[{\hat{\psi }}_{\mu }^{\left(1\right)},{\hat{\psi }}_{\mu }^{\left(2\right)},\cdots ,{\hat{\psi }}_{\mu }^{\left(N\right)}\right]\\ \Omega_{\mu }=\mathrm{diag}\left[\omega_{\mu }^{\left(1\right)},\omega_{\mu }^{\left(2\right)},\cdots ,\omega_{\mu }^{\left(N\right)}\right]. \end{array}\right.$$

${\hat{\psi }}_{\mu }^{N}$ represents the natural vibration mode, and $\omega_{\mu }^{N}$ is the corresponding eigenfrequency. The derivation process is given in Appendix B.2.

#### 2.3.3. Band-Gap Analysis through the Two Subsystems

The average value of elements on the main diagonal of the effective pseudo-density matrix $R^{e}$ is the effective mass density. According to Equation (17), the values of elements on the main diagonal of $\tilde{R}$ approach negative infinity as ω approaches $\omega^{*}$ from the right, and $R^{e}$ obviously follows the same trend.

From Equation (19), it can be found that when ω approaches $\omega_{\mu }$,

(21) $$\Psi_{\mu }{\Psi_{\mu }}^{T}N_{\mu }^{T}\beta =0.$$

With

(22) $$\left\{\begin{array}{c} \beta =R^{e}\ddot{u}\\ \Psi_{\mu }{\Psi_{\mu }}^{T}N_{\mu }^{T}\neq 0, \end{array}\right.$$

so the effective, frequency-dependent pseudo-density $R^{e}$ is 0.

With the above work, consider the frequency range $\left[\omega_{\mu }^{*},\omega_{\mu }\right]$ determined by the natural frequencies of the restricted and unrestricted subsystems. Within this frequency range, $R^{e}$ initially is unbounded and negative-definite, eventually evolving to 0 at the end of the range. Therefore, $\left[\omega_{\mu }^{*},\omega_{\mu }\right]$ can be used to determine the band gap of LRAMs [39].

**Figure 3:**
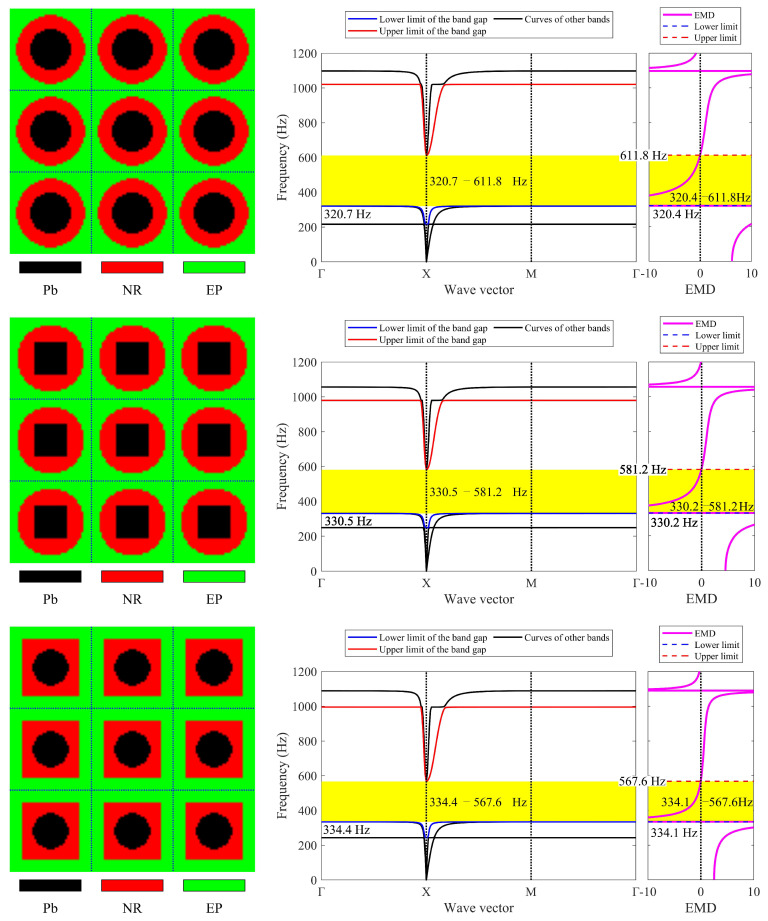
Different LRAM unit cells with corresponding dispersion curves and effective mass density curves.

Figure 3 presents the dispersion curves and effective mass density (EMD) curves obtained from different LRAMs. The band gaps shown in the two types of curves are compared to demonstrate that the simplified band-gap calculation method is feasible. The lattice size of the LRAM cell is 0.02 × 0.02 m. The design domains presented in the figures contain 3 × 3 cells. The materials utilized include lead (Pb), rubber (NR), and epoxy (EP), with their corresponding material parameters detailed in Table 1.

In the dispersion curves in Figure 3, the yellow zone denotes the band gap determined by the bands. In the EMD curves in Figure 3, the yellow zone representing the band gap is determined by the eigenfrequencies of the restricted and unrestricted subsystems. As observed in the dispersion curves and EMD diagrams, the yellow zones representing the first band gap only show a difference at the lower edge, with a value of less than half a percent (less than 1 Hz). Thus, this simplified calculation method can be utilized in the optimization process. Since the simplifying assumptions are more valid as the system approaches a quasistatic situation, this paper only studies the optimization problem of the first band gap.

## 3. A Multi-Material Optimization Method for LRAMs

A level-set-based method is introduced to address LRAM band-gap optimization problems using multiple materials in this work. The compactly supported radial basis function (CSRBF) is employed in the development of the PLSM [54].

### 3.1. The Uniform Multi-Material Description Model

Inspired by the “color” level-set model [26] and the uniform multiphase materials interpolation (UMMI) method [34], a level-set-based uniform multiphase description model is introduced. In this model, each material, except for the matrix material, is denoted by a similar combination form that utilizes level-set functions, as shown below

(23) χk=H(ϕk)∏i=1i≠kp−1(1−H(ϕi)),k≠pχk=1−∑i=1p−1χi,k=p,

where $\chi_{k}$ represents the *k*th material and *p* is the total number of material phases. Using p=3 as an illustration, two variables are employed to represent three materials within the design domain

(24) $$\left\{\begin{array}{c} \chi_{1}=H\left(\phi_{1}\right)\left(1-H\left(\phi_{2}\right)\right)\\ \chi_{2}=H\left(\phi_{2}\right)\left(1-H\left(\phi_{1}\right)\right)\\ \chi_{3}=1-H\left(\phi_{1}\right)\left(1-H\left(\phi_{2}\right)\right)-H\left(\phi_{2}\right)\left(1-H\left(\phi_{1}\right)\right), \end{array}\right.$$

where *H* is the smeared Heaviside function

(25) $$H\left(\phi \right)=\left\{\begin{array}{c} 0,\;\;\;\;\;\;\;\;\;\;\phi <-\Delta \\ \frac{3}{4}\left(\frac{\phi }{\Delta }-\frac{\phi^{3}}{3\Delta^{3}}\right)+\frac{1}{2},\;-\Delta \leq \phi \leq \Delta \\ 1,\;\;\;\;\;\;\;\;\;\;\phi >\Delta , \end{array}\right.$$

where Δ specifies the breadth of the numerical approximation of *H*.

**Figure 4:**
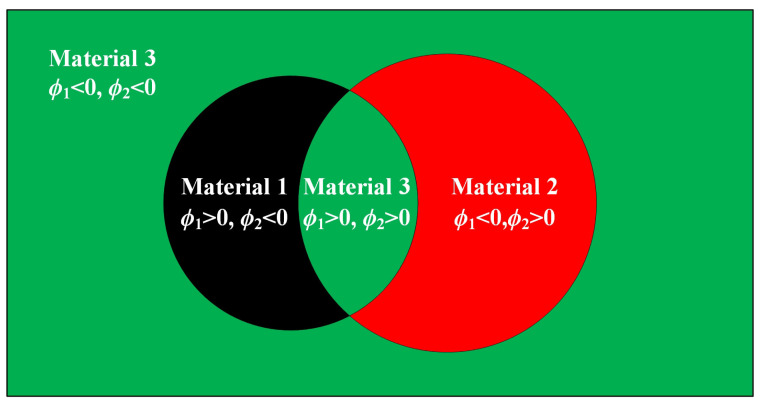
The description model involves representing three materials using two level-set functions: $\phi_{1}$ and $\phi_{2}$.

To be more illustrative, take Figure 4 as an example to illustrate the description model. Three materials are represented by two level-set functions in this illustration.

As demonstrated in Equation (23), when representing materials apart from the matrix material, the respective level-set function takes on a value of 1, whereas the remaining level-set functions are set to 0. This implies that the material type can only change to another type when one level-set function changes from 1 to 0, another changes from 0 to 1, and the retained ones remain unchanged. Otherwise, the material type will change to the matrix material. The mutual exclusivity of material descriptions makes direct conversion between materials other than the matrix material less smooth in the presented model. According to our numerical experience, this is beneficial for LRAM band-gap optimization. One possible reason is that the coating’s stiffness is significantly lower by several orders of magnitude compared to that of the scatterer. This means that direct conversion between the coating and the scatterer may lead to a relatively large change in the objective value.

### 3.2. The CSRBF-Based Parameterized Level-Set Method

According to existing research [22,23], variables in LSMs are updated using the Hamilton–Jacobi partial differential equation (PDE) given below:

(26) $$\frac{\partial \phi }{\partial t}-V_{q}\left|\nabla \phi \right|=0,$$

where *t* represents pseudo-time, $V_{q}=V\cdot \left(-\frac{\nabla \phi }{\left|\nabla \phi \right|}\right)$ denotes the normal velocity toward the outside, and ∇(·) represents the gradient.

By decomposing the original Hamilton–Jacobi PDE into a system of coupled ordinary differential equations (ODEs) using CSRBFs, the method known as the parameterized level-set method is proposed [54,55]. The level-set function ϕ(x,t) is interpolated by *n* CSRBFs, which can be given as

(27) $$\phi \left(t\right)=g\left(x\right)\alpha \left(t\right)=\sum_{i=1}^{n}g_{i}\left(x\right)\alpha_{i}\left(t\right),$$

where the design variable $\alpha \left(t\right)=[\alpha_{1}(t),\alpha_{2}(t),\ldots ,\alpha_{n}(t)]$ consists of expansion coefficients that vary with pseudo-time *t*. $g\left(x\right)=[g_{1}(x),g_{2}(x),\ldots ,g_{n}(x)]$ is the vector of CSRBFs.

This work adopts CSRBFs with C2 continuity. At the specified x, $g_{i}\left(x\right)$ with *r* as the support radius is given as follows:

(28) $$g_{i}\left(x\right)=\max{\left\{0,\left\{1-r\right\}\right\}}^{4}\left(4r+1\right).$$

Then, Equation (26) transforms into the following governing equation

(29) $$g\left(x\right)\frac{d\alpha (t)}{dt}-V_{q}\left|\left(\nabla g(x))\alpha (t\right)\right|=0,$$

where

(30) $$\left|(\nabla g(x))\alpha \right|=\sqrt{{\left(\frac{\partial g(x)}{\partial x}\alpha \right)}^{2}+{\left(\frac{\partial g(x)}{\partial y}\alpha \right)}^{2}}.$$

The optimization proceeds by updating α(t) until a convergence result is achieved.

### 3.3. Mathematical Model for LRAM Band-Gap Optimization Problems with Multiple Materials

Based on the work described above, multi-material band-gap optimization models for LRAMs with different objectives and constraints are built. In these models, the natural frequencies of the two subsystems established in Section 2 are utilized to determine the LRAM band gap.

#### 3.3.1. Maximizing the Band-Gap Width While Ensuring It Encompasses a Specified Frequency Range

Obtaining a wider band gap at a lower frequency is significant for applications. This is typically the objective of LRAM band-gap optimization. However, in certain cases, the band gap of the LRAM obtained should encompass a specified frequency range to isolate the vibration or noise as required. The optimization model for maximizing the band-gap width while encompassing a specified frequency range is established as follows:

(31) $$\begin{array}{c} \;\mathrm{Max}:f=\frac{\omega_{\mu }-\omega_{\mu }^{*}}{\omega_{\mu }^{*}}\\ \;\mathrm{Find}:\;\alpha_{i=1,2,\ldots ,m-1}^{j=1,2,\ldots ,N}\\ \;S.t.\;\;:\;({\hat{K}}_{\mu 2}^{*}-\omega_{\mu }^{*2}{\hat{M}}_{\mu 2}^{*}){\hat{\psi }}_{\mu 2}^{*}=0\\ \;\;\;\;({\hat{K}}_{\mu }-\omega_{\mu }^{2}{\hat{M}}_{\mu }){\hat{\psi }}_{\mu }=0\\ \;\;\;\;\omega_{\mu }^{*}<\omega_{0}-\omega_{r}\\ \;\;\;\;\omega_{\mu }>\omega_{0}+\omega_{r} \end{array}$$

where *N* denotes the finite-element quantity, *m* represents the number of material phases, $\omega_{0}$ represents the center frequency, and $\omega_{r}$ denotes the deviation of the band-gap boundaries from the center frequency. These parameters are utilized to specify the required frequency range.

#### 3.3.2. Obtaining Light LRAMs with a Specified Band Gap

To precisely control the propagation of vibrations or sound waves, optimization problems aimed at obtaining LRAMs with a specified band gap are also studied. One significant application of these LRAMs is in filter design. Meanwhile, achieving a lighter structure is a crucial objective considered in the design process. Here, the optimization model for obtaining light LRAMs with a specified band gap is established.

(32) $$\begin{array}{c} \;\mathrm{Min}:f=\sum_{i=1}^{m}\rho_{i}V_{i}\\ \;\mathrm{Find}:\;\alpha_{i=1,2,\ldots ,m-1}^{j=1,2,\ldots ,N}\\ \;S.t.\;\;:\;({\hat{K}}_{\mu 2}^{*}-\omega_{\mu }^{*2}{\hat{M}}_{\mu 2}^{*}){\hat{\psi }}_{\mu 2}^{*}=0\\ \;\;\;\;({\hat{K}}_{\mu }-\omega_{\mu }^{2}{\hat{M}}_{\mu }){\hat{\psi }}_{\mu }=0\\ \;\;\;\;\omega_{o1}-\omega_{r1}<\omega_{\mu }^{*}<\omega_{o1}+\omega_{r1}\\ \;\;\;\;\omega_{o2}-\omega_{r2}<\omega_{\mu }<\omega_{o2}+\omega_{r2} \end{array}$$

where the same parameters have the same meanings as those in the mathematical model in Equation (31). $\rho_{i}$ is the mass density, and $V_{i}$ is the volume fraction. $\omega_{o1}$ and $\omega_{o2}$ are the target frequencies of $\omega_{\mu }^{*}$ and $\omega_{\mu }$, respectively. According to our numerical experience, it may be difficult to achieve convergence when setting the lower and upper limits of the band gap at specified values. So, in implementation, the lower and upper frequency constraints will be set as certain frequency ranges. $\omega_{r1}$ is used to represent the deviation of $\omega_{\mu }^{*}$ from $\omega_{o1}$, and $\omega_{r2}$ is used to represent the deviation of $\omega_{\mu }$ from $\omega_{o2}$. Take the specified target frequency range of 500–700 Hz as an example, the deviation of the obtained frequencies from the specified constraint frequencies is permitted within a range of ten percent. $\omega_{o1}$ is 500 Hz and $\omega_{o2}$ is 700 Hz. $\omega_{r1}$ and $\omega_{r2}$ are obtained as 0.1×(0.5×(700−500))=10 Hz. So, $\omega_{\mu }^{*}$ ranges from 490 to 510 Hz, and $\omega_{\mu }$ ranges from 690 to 710 Hz.

### 3.4. Sensitivity Analysis

As the method presented is gradient-based, conducting sensitivity analysis is necessary in the optimization process. The obtained sensitivity information is used to determine the search direction.

The sensitivity information for the objective described in Equation (31) with respect to χ can be calculated as follows:

(33) $$\frac{\partial f(\chi )}{\partial \chi }=\frac{\omega_{\mu }^{*}\frac{\partial \omega_{\mu }}{\partial \chi }-\omega_{\mu }\frac{\partial \omega_{\mu }^{*}}{\partial \chi }}{{\omega_{\mu }^{*}}^{2}}.$$

Normalizing the eigenvectors to the global mass matrix, one can obtain the sensitivities of eigenfrequencies ω by

(34) $$\frac{\partial \omega }{\partial \chi }=\frac{1}{2\omega }\Psi^{T}\left(\frac{\partial K}{\partial \chi }-\omega^{2}\frac{\partial M}{\partial \chi }\right)\Psi \;.$$

The sensitivity information for the objective described in Equation (32) with respect to χ can be calculated as follows:

(35) $$\frac{\partial f\left(\chi_{i}\right)}{\partial \chi_{i}}=\sum_{i=1}^{m}\rho_{i}\frac{\partial V_{i}}{\partial \chi_{i}}=\sum_{i=1}^{m}\rho_{i}v^{0},$$

where $\chi_{i}$ denotes the *i*th material. $v^{0}$ is a vector whose elements denote the volume fraction of solid finite elements.

The derivative of H(ϕ) is the Dirac function, given by

(36) $$\delta \left(\phi \right)=\left\{\begin{array}{c} \frac{3}{4\Delta }\left(1-\frac{\phi^{2}}{\Delta^{2}}\right),\;\left|\phi \right|\leq \Delta \\ 0,\;\;\;\;\;\;\;\;\;\left|\phi \right|>\Delta . \end{array}\right.$$

Considering Equations (27) and (36), the sensitivity of *f* is given as

(37) $$\frac{\partial f}{\partial \alpha }=g{\left(x\right)}^{-1}\left(\frac{\partial f}{\partial \chi }\frac{\partial \chi }{\partial (H(\phi ))}\delta \left(\phi \right)\right).$$

Further details about the sensitivity of K and M are presented in Appendix C.

Note that the sensitivity analysis shown above is limited to the problem with a single eigenvalue. When the system has multiple eigenvalues, the sensitivity information should be calculated using the method presented in [56,57,58].

This work adopts the method of moving asymptotes (MMA) [59] to update the design variables.

## 4. Results and Discussion

**Figure 5:**
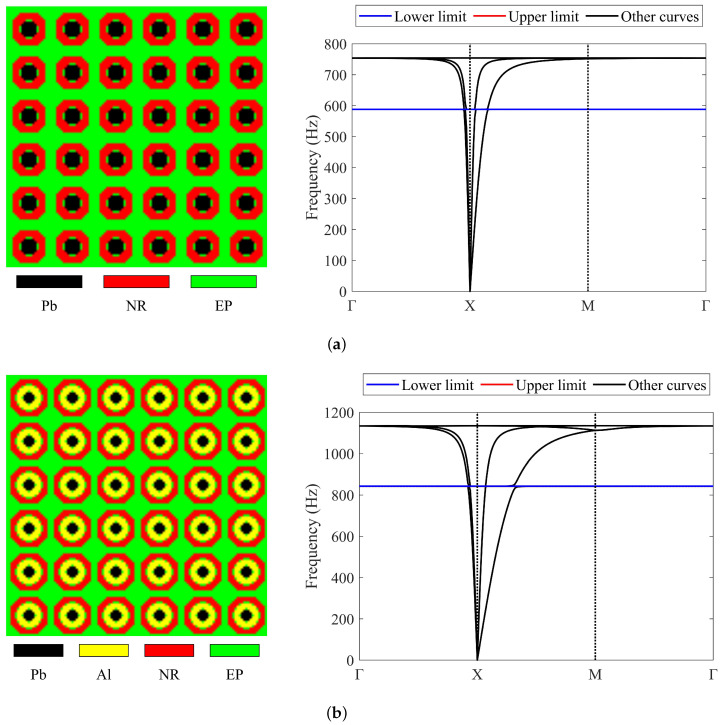
Initial guesses and corresponding band diagrams of numerical cases in Section 4.1 and Section 4.2 (in the band diagrams, the blue and red lines overlap due to the absence of band gaps): (**a**) three materials; (**b**) four materials.

This section presents several 2D three-material and four-material numerical cases to confirm the feasibility and utility of the approach. The impact of the initial guesses on the optimized results is also reported. The square unit cell studied has square symmetry [16,60,61], exhibiting mirror symmetry along the horizontal, vertical, and two diagonal lines. The lattice size of the studied LRAM cell is 0.02×0.02m. The results presented in the topology figures contain 3 × 3 cells. All calculations were conducted using MATLAB R2020b. The materials used include lead, aluminum, rubber, and epoxy, with their material parameters detailed in Table 1. The MMA parameters used are $a_{0}=1$, a=1, c=1000, d=1, and move=0.1. The meaning of these parameters can be found in Svanberg’s paper [62]. In these numerical examples, only the band gap between the third and fourth bands is studied. The initial guesses and corresponding band diagrams are presented in Figure 5, unless otherwise specified. Obviously, there are no band gaps in the initial designs. It should be pointed out here that the final band diagrams presented in all numerical cases are calculated using the conventional method based on the boundary of the irreducible Brillouin zone. In all band diagrams in this section, black vertical dotted lines are used to divide different boundary ranges of the irreducible Brillouin zone, and blue and red horizontal dotted lines are used to indicate the band gap zones.

### 4.1. Numerical Examples of Maximizing the Band-Gap Width While Ensuring It Encompasses a Specified Frequency Range

In this subsection, numerical examples of maximizing the band-gap width encompassing a specified frequency range are presented. In the three-material cases, lead, rubber, and epoxy are utilized. In the four-material examples, lead, aluminum, rubber, and epoxy are employed.

#### 4.1.1. Numerical Examples Where the Obtained Band Gaps Should Encompass 400–600 Hz

**Figure 6:**
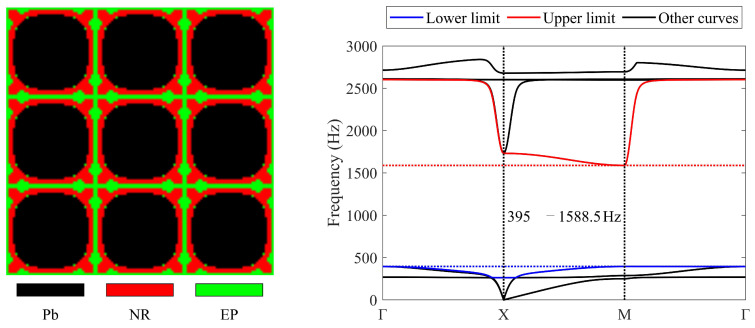
Optimized solution and corresponding band diagram (the band gap should encompass 400–600 Hz) obtained with a mesh resolution of 40 × 40.

First, three materials are used to obtain LRAMs with the required band gaps. The design domain is partitioned into linear four-node elements with a resolution of 40 × 40. Figure 6 presents the obtained topology and corresponding band diagram.

In the optimized results, the black region is lead, rubber is depicted in red, and the green region corresponds to epoxy. The scatterer is made of lead, which has a high mass density, while the coating is made of rubber, a soft material. This is in line with the band-gap formation mechanism in LRAMs, as described in the existing literature [1,2,18,63,64].

**Figure 7:**
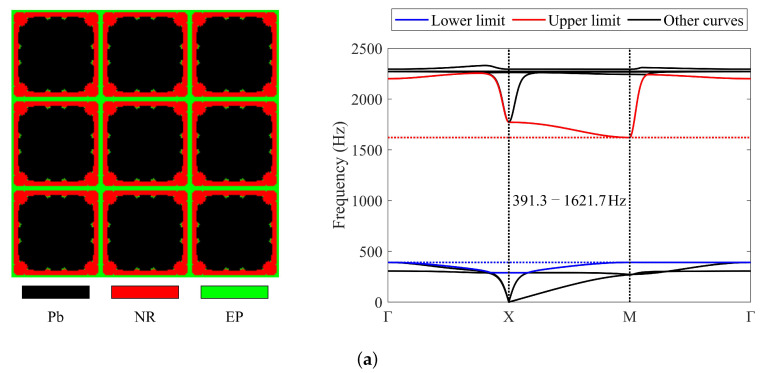

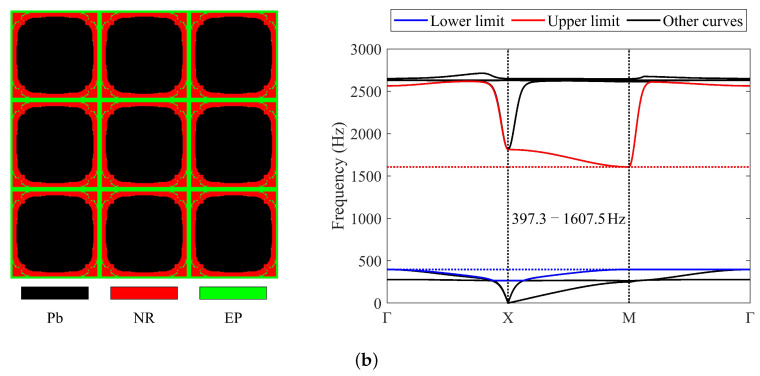
Optimized solutions and corresponding band diagrams (the band gap should encompass 400–600 Hz) obtained with different mesh resolutions: (**a**) 80 × 80; (**b**) 160 × 160.

To investigate the impact of different mesh resolutions on the final results, mesh resolutions of 80 × 80 and 160 × 160 are also employed for the optimization. Figure 7 depicts the obtained results.

The band gaps of the optimized topologies in Figure 6 and Figure 7a,b are 395–1588.5 Hz, 391.3–1621.7 Hz, and 397.3–1607.5 Hz, respectively. The band gaps determined by the proposed simplified method are 394.3–1588.5 Hz, 390.9–1621.7 Hz, and 396.6–1607.5 Hz. It can be observed that only slight differences appear at the lower limits. The objective values are 3.02, 3.14, and 3.05, respectively. In the three resulting topologies, lead occupies 0.63, 0.66, and 0.67 of the total volume, and rubber occupies 0.24, 0.23, and 0.22 of the total volume. The remaining region is occupied by epoxy. These results indicate that the mesh resolution impacts the final optimized topology. However, with the same objectives and constraints, the resulting topologies will be similar, and their band gaps will meet the specified frequency constraints while being wide enough. Since the numerical examples aim to verify the feasibility of the presented approach, a resolution of 40 × 40 is used in the remaining examples.

**Figure 8:**
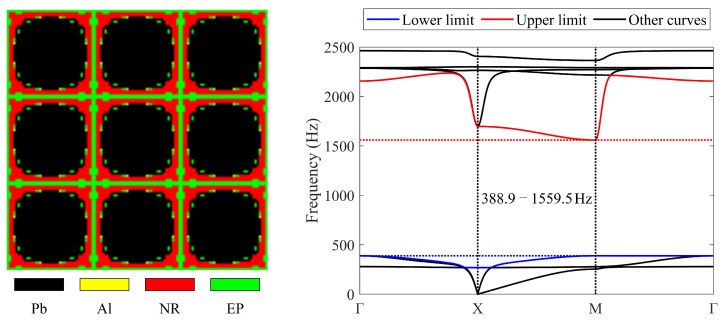
Optimized solution and corresponding band diagram (the band gap should encompass 400–600 Hz) obtained using four materials.

**Figure 9:**
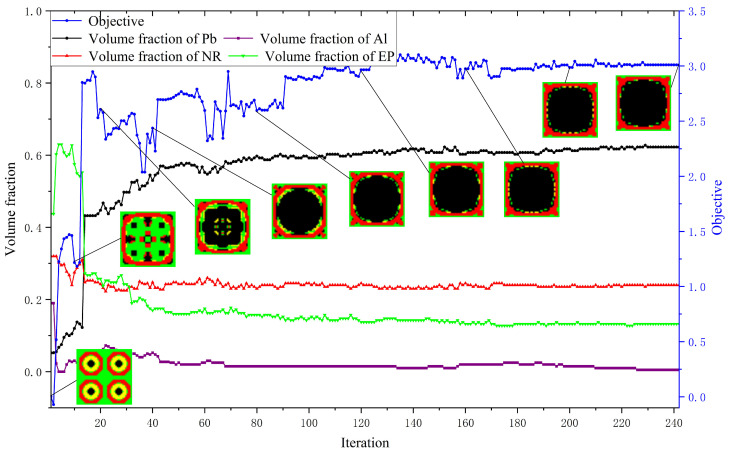
Iteration histories of the topology, objective, and volume fractions of the four-material case (the band gap should encompass 400–600 Hz).

Four materials are also used to obtain the required LRAM under the same objective and constraints. The obtained topology and band diagram are presented in Figure 8. Figure 9 illustrates the evolutionary history of the four-material case. In the obtained topology, black, yellow, red, and green denote lead, aluminum, rubber, and epoxy, respectively.

The band gap of the optimized topology is 388.9–1559.5 Hz, which is also very close to 388.3–1559.5 Hz, as determined by the simplified calculation method. The objective value is 3.01. The final volume fractions of lead, aluminum, rubber, and epoxy are 0.6225, 0.005, 0.24, and 0.1325, respectively. Due to changes in material distribution and the band gap that appeared, the objective value experienced acute changes in the initial iteration steps of the optimization. Subsequently, the optimization converges, meeting the frequency constraints. As shown in Figure 9, the aluminum contained in the initial guess almost disappears (decrease to 0.005) in the obtained topology. According to the band-gap formation mechanism [1,2,18,63,64], LRAMs can be composed of scatterers, coatings, and matrices. The scatterer is composed of a material with a high mass density, while the coating is composed of a soft material. Once the coating is determined, the band-gap width is influenced by the difference in mass density between the matrix and scatterer. Furthermore, the higher the mass density of the scatterer and the softer the coatings, the lower the initial frequency of the band gap. The mass density of lead is much higher than that of aluminum. Therefore, it is easier to achieve a low-frequency band gap with a better objective value using lead as the scatterer instead of aluminum. This is a possible reason why the volume fraction of aluminum decreases and is substituted by lead.

#### 4.1.2. Numerical Examples Where the Obtained Band Gaps Should Encompass 1000–2000 Hz

**Figure 10:**
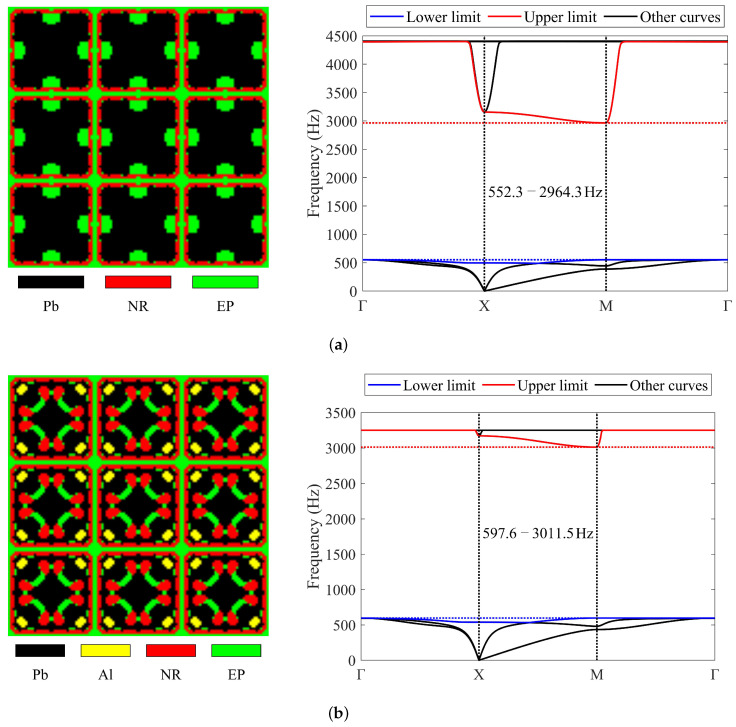
Optimized solutions and corresponding band diagrams (the band gap should encompass 1000–2000 Hz) obtained using three or four materials: (**a**) three-material case; (**b**) four-material case.

In this subsection, three-material and four-material examples where the obtained band gaps should encompass the frequency range of 1000–2000 Hz are presented. Figure 10 presents the topologies obtained and their corresponding band diagrams. In the optimized results, black, yellow, red, and green represent lead, aluminum, rubber, and epoxy, respectively.

The final band gaps of the three-material and four-material cases are 552.3–2964.3 Hz and 597.6–3011.5 Hz, respectively. The band gaps determined by the simplified method are 552.1–2964.3 Hz and 597.4–3011.5 Hz, with only slight differences at the lower limits. In the three-material case, lead, rubber, and epoxy account for 0.65, 0.15, and 0.2 of the final design in volume, respectively. In the four-material case, lead, aluminum, rubber, and epoxy occupy 0.48, 0.04, 0.28, and 0.2 of the final design in volume, respectively. The objective values of the two obtained results are 4.37 and 4.04, and the masses are 3.17 and 2.52, respectively. In the four-material case, the final objective value decreased by 7.6% compared to the three-material case, while the mass of the structure decreased by 20.4%.

Section 4.1.1 and Section 4.1.2 show that the method successfully obtains LRAMs with sufficiently wide band gaps encompassing the specified frequency ranges. This demonstrates the method’s effectiveness in using multiple materials to solve band-gap optimization problems in LRAMs. It can be seen from the numerical examples of three and four materials that using more types of materials in optimization does not necessarily result in a wider band gap. This is because LRAM band gaps are influenced by many factors, including material properties, topology shapes, symmetries, filling fractions, and more. However, when the lattice size is specified, material properties become the predominant factor in determining LRAM band gaps. In the four-material examples, since a certain volume of lead is substituted with aluminum, it can be difficult to obtain a wider band gap compared to the three-material examples under certain circumstances due to differences in the parameters of the two materials. But using more kinds of materials to design LRAMs can obtain results that meet more goals and constraints. For example, in Section 4.1.2, the addition of aluminum in the four-material case results in a lighter structure compared to the three-material case, with a much smaller decrease in the band-gap width relative to the mass. In Table 2, a comparison is presented to show that a lighter design can be obtained while similar performance is achieved using more materials.

### 4.2. Numerical Examples of Obtaining Light LRAMs with a Specified Band Gap

In this section, numerical examples of obtaining light LRAMs with a specified band gap are presented. Following the rule mentioned in Section 3.3.2, the band gaps of the optimized results are set as 500 ± 10–700 ± 10 Hz, 700 ± 10–900 ± 10 Hz, 800 ± 40–1600 ± 40 Hz, and 700 ± 90–2500 ± 90 Hz, respectively, regardless of whether they are three-material or four-material cases.

#### 4.2.1. Three-Material Cases

**Figure 11:**
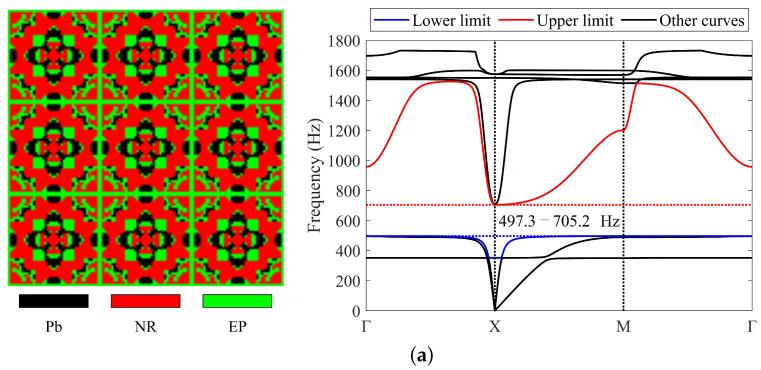

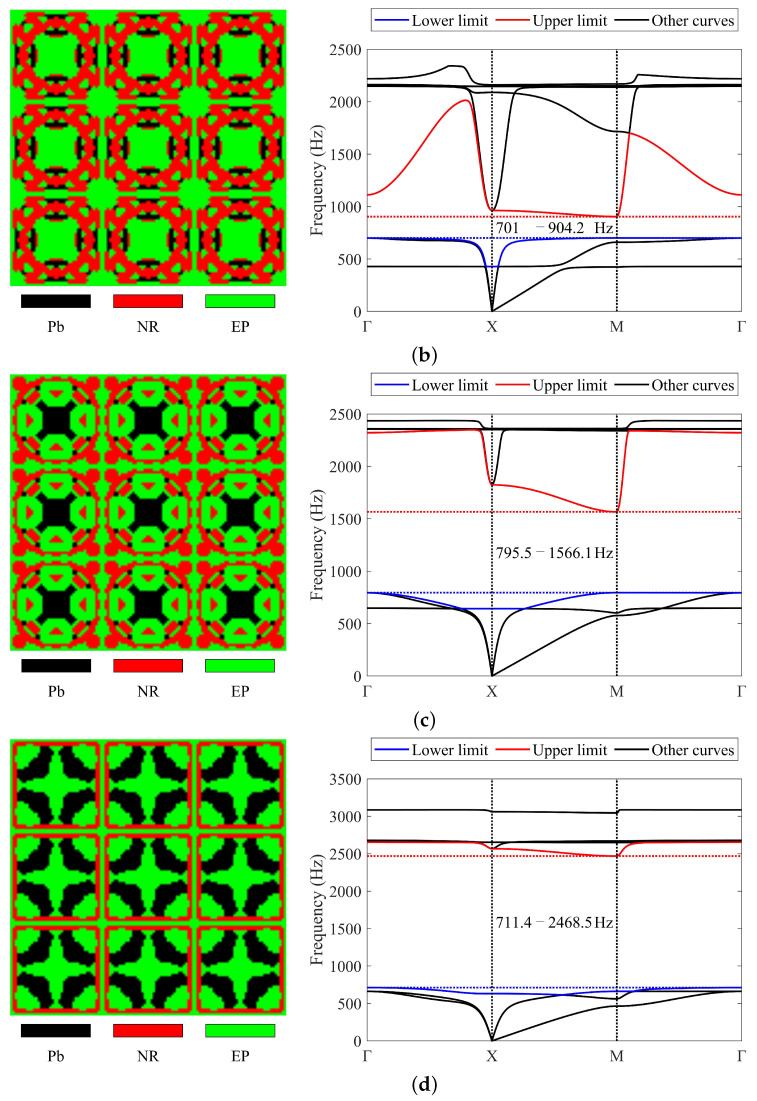
Optimized solutions and corresponding band diagrams for three-material cases with different specified band gaps: (**a**) 500 ± 10–700 ± 10 Hz; (**b**) 700 ± 10–900 ± 10 Hz; (**c**) 800 ± 40–1600 ± 40 Hz; (**d**) 700 ± 90–2500 ± 90 Hz.

Lead, rubber, and epoxy are utilized to obtain the required LRAMs in these cases. The optimized results are shown in Figure 11. In cases (a) to (d), lead occupies 0.17, 0.1, 0.14, and 0.34 of the total volume, while rubber occupies 0.52, 0.42, 0.33, and 0.16, respectively. The remaining region is occupied by epoxy. The masses of these obtained structures are 1.22, 0.92, 1.07, and 1.89, respectively. The band gaps of the optimized topologies are 497.3–705.2 Hz, 701–904.2 Hz, 794.4–1566.1 Hz, and 711.4–2468.5 Hz, respectively. The band gaps determined by the proposed simplified method are also provided, which are 496.1–705.2 Hz, 699.9–904.2 Hz, 795.5–1566.1 Hz, and 711.1–2468.5 Hz, respectively. Figure 11 shows that LRAMs with the specified band gaps are successfully obtained. The proposed method has the potential to design devices with specified functions, such as filters.

#### 4.2.2. Four-Material Cases

**Figure 12:**
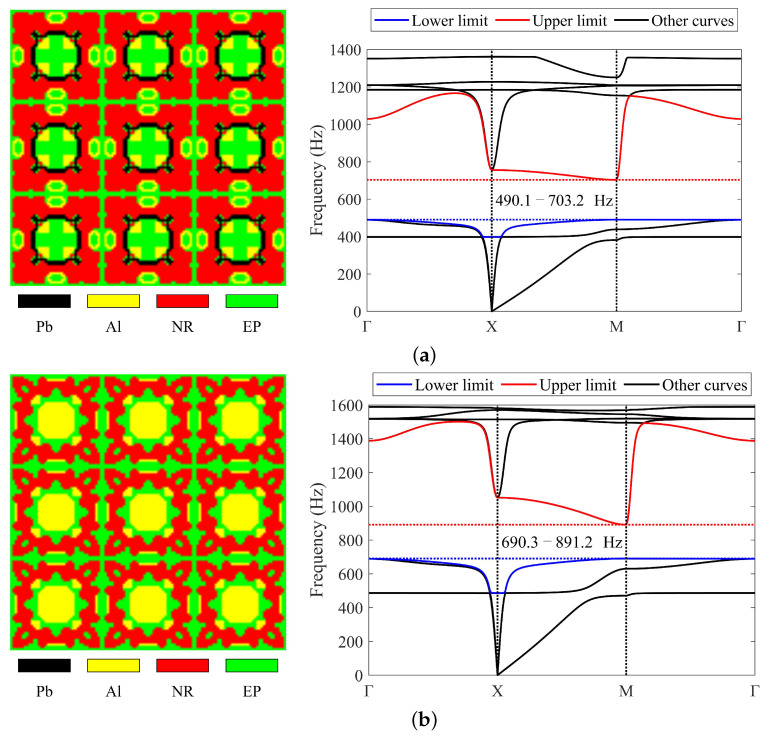

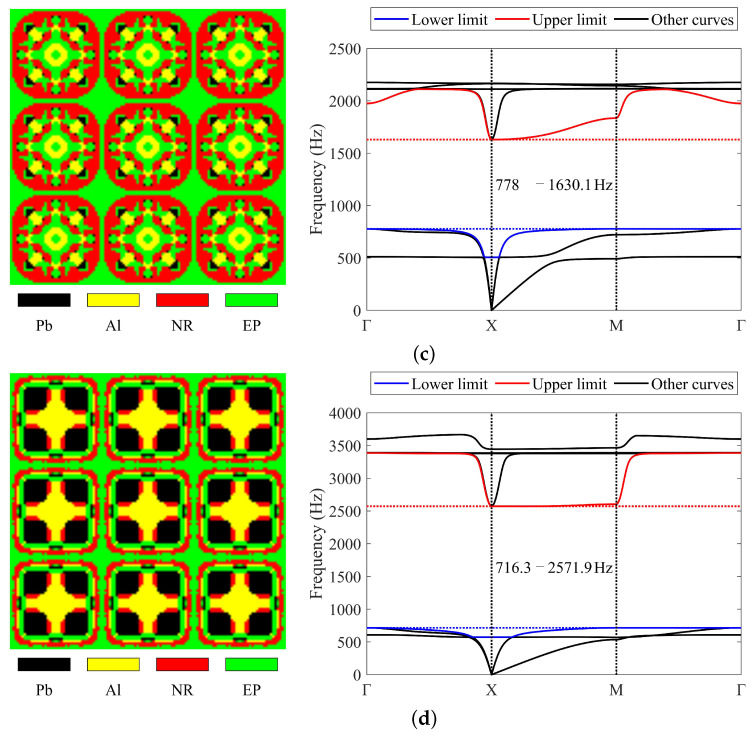
Optimized solutions and corresponding band diagrams for four-material cases with different specified band gaps: (**a**) 500 ± 10–700 ± 10 Hz; (**b**) 700 ± 10–900 ± 10 Hz; (**c**) 800 ± 40–1600 ± 40 Hz; (**d**) 700 ± 90–2500 ± 90 Hz.

Lead, aluminum, rubber, and epoxy are utilized to obtain the required LRAMs in these cases. The optimized results are shown in Figure 12. In case (a), lead, aluminum, rubber, and epoxy occupy 0.08, 0.1, 0.52, and 0.3 of the total volume, respectively. In case (b), these values are 0, 0.2, 0.46, and 0.34. In case (c), these values are 0.03, 0.11, 0.43, and 0.43. In case (d), these values are 0.27, 0.21, 0.21, and 0.31, respectively. The masses of these obtained structures are 0.89, 0.61, 0.71, and 1.73, respectively. The band gaps of the optimized topologies are 490.1–703.2 Hz, 690.3–891.2 Hz, 778–1630.1 Hz, and 716.3–2571.9 Hz, respectively. The band gaps determined by the proposed simplified method are also provided, which are 489.6–703.2 Hz, 689–891.2 Hz, 777.4–1630.1 Hz, and 715.5–2571.9 Hz, respectively.

The results depicted in Figure 12 demonstrate that this approach can also employ four materials to obtain LRAMs with a specified band gap. In this optimization problem, whether using three or four materials, the final band gaps and those determined by the proposed simplified method show differences of less than half a percent (less than 2 Hz), also only at the lower limits of the band gaps. This demonstrates that the simplified band-gap calculation method can be applied to the optimization process.

With more kinds of materials providing more design possibilities, multi-material topology optimization methods may have the ability to achieve results that meet more objectives and constraints. Since structural mass is an important factor that impacts the structure’s application, it should be taken into consideration during the design stage. Table 2 presents a comparison of the changes in the band gaps and masses of the resulting topologies using three or four materials described in Section 4.2.

Table 2 shows that compared to using three materials, lighter designs with the required band gaps can be obtained using four materials. While this conclusion may not be general, it demonstrates the possibility of utilizing a greater variety of materials to design lighter LRAMs without significantly altering the band-gap width. Therefore, this multi-material approach is capable of obtaining LRAMs with the required band gap while also reducing their mass.

### 4.3. The Impact of Initial Guesses on the Final Optimized Results

To investigate the impact of the initial guess on the final result, numerical examples with different initial guesses are provided in Table 3. The objective of these examples is to obtain LRAMs with the widest band gap while ensuring that the band gap encompasses the specified frequency range of 400–600 Hz. In these cases, if the initial guess is not asymmetrical, the method described in Dong et al.’s paper [65] is used to calculate the band gap.

Table 3 shows that different initial guesses can result in different final topologies. This indicates that numerous locally optimal results exist in multi-material LRAM band-gap optimizations. However, if all the materials are sufficiently discretely distributed in the initial guesses, topologies with band gaps that fulfill the objectives and constraints can be obtained. As a result, the proposed method can significantly assist in designing LRAMs with the required band gaps.

As observed from the numerical examples in this section, the proposed method can use multiple materials to obtain LRAMs with specified band gaps. This method can be employed to design metamaterials that attenuate vibration and noise as required. However, challenges remain that affect its practical application, such as selecting materials for specific applications and incorporating the impact of manufacturing uncertainty into the optimization process.

## 5. Conclusions

In this paper, based on the homogenization framework, a level-set-based multi-material topology optimization method is proposed for designing LRAMs with specified frequency constraints. The method has the following features:

(a) A simplified calculation method for LRAM band gaps is established using the homogenization framework. This method utilizes a restricted subsystem and an unrestricted subsystem to determine the LRAM band gap.

(b) In the presented uniform level-set-based multi-material description model, each material, except for the matrix material, is denoted by a similar combination formulation of level-set functions. This reduces direct conversion between materials other than the matrix material.

(c) The presented multi-material optimization method can obtain LRAMs with band gaps that fulfill specified frequency constraints. The two problems of obtaining LRAMs with the maximum band-gap width while ensuring that this band gap encompasses a specified frequency range, and obtaining light LRAMs with a specified band gap, are tested using multiple materials.

The results of numerical cases using three or four materials confirm the feasibility and effectiveness of the presented method in obtaining LRAMs with the required band gaps. Compared with similar optimization strategies presented by Roca et al. [5], the method proposed here removes limitations such as considering the matrix fixed frame as infinitely stiff and the coating material as massless. Moreover, the presented method does not restrict the analysis to a single dimension and can obtain LRAMs with the required band gaps using multiple materials. This method can obtain LRAMs with wide low-frequency band gaps based on the local resonance mechanism. Thus, it shows great promise for attenuating specified frequency spectra as required, such as mechanical vibrations or environmental noise.

The proposed method can use multiple materials to solve LRAM optimization problems, potentially achieving results that meet more objectives and constraints. For instance, this work shows that this approach is capable of using a greater variety of materials to obtain LRAMs with the required band gaps while simultaneously reducing their mass. However, it should be mentioned that due to the limitations of simplifying assumptions, when the system approaches the quasistatic case, the assumed simplifications hold more strongly. As the frequency increases, the simplified band-gap calculation method may become less accurate as the separation of scales approaches its limits. However, according to our numerical experience, in most first-band-gap optimization problems, the proposed method can obtain the required LRAMs that meet objectives and constraints. Additionally, higher-frequency band gaps can be easily obtained by optimizing LRAMs with a smaller lattice size.

Multi-material topology optimization is often criticized. One reason is that the obtained results face challenges in properly addressing interfaces between different materials in the manufacturing process. The fabricated metamaterials should ensure that the different materials (where material parameters can be significantly different) are properly connected, with minimal defects and a high degree of precision. Three-dimensional printing technology may help facilitate practical applications. Additionally, the impact of material interfaces and manufacturing uncertainty is not considered in this work. These aspects will be explored in our future research. Applying the method to solve three-dimensional and large-scale problems, as well as designing other types of metamaterials, will also be studied in future work.

## Figures and Tables

**Table 1:** Parameters of materials utilized to obtain LRAMs.

	Mass Density ρ ($\mathrm{kg}/m^{3}$)	Lame Constant λ (GPa)	Shear Modulus μ (GPa)
Lead (Pb)	11,600	42	14.9
Aluminum (Al)	2700	51.1	26.3
Rubber (NR)	1300	6.05 × $10^{-4}$	4 × $10^{-5}$
Epoxy (EP)	1180	3.74	1.31

**Table 2:** The changes in the band gap and mass from three-material to four-material cases.

Numerical Case	Band-Gap Range (Three-Material Case)	Band-Gap Range (Four-Material Case)	The Change in Band-Gap Width	The Change in Mass
Case 1 (500 ± 10–700 ± 10 Hz)	497.3–705.2 Hz	490.1–703.2 Hz	2.5%	−26.6%
Case 2 (700 ± 10–900 ± 10 Hz)	701–904.2 Hz	690.3–891.2 Hz	−1.1%	−33.2%
Case 3 (800 ± 40–1600 ± 40 Hz)	795.5–1566.1 Hz	778–1630.1 Hz	10.6%	−33.9%
Case 4 (700 ± 90–2500 ± 90 Hz)	711.4–2468.5 Hz	716.3–2571.9 Hz	5.6%	−8.5%

**Table 3:** The results obtained with different initial guesses.

Initial Guess	Total Number of Iterations	Topology	Band-Gap Range (Hz)
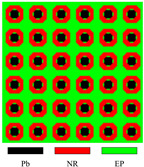	133	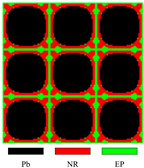	395–1588.5
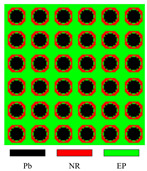	329	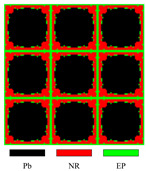	390.9–1568
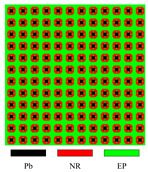	96	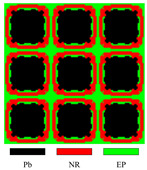	370.9–1459.1
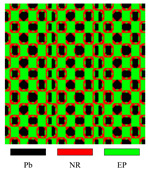	116	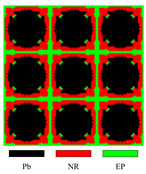	353–1443.8

## Data Availability

The raw data supporting the conclusions of this article will be made available by the authors on request.

## Appendix A. The Establishment of the Restricted Subsystem

For 2D cases, through Equations (4) and (5), the local macroscale homogenized force β can be calculated as

(A1) $$\beta =I^{T}\left({\hat{M}}_{\mu }{\ddot{\hat{U}}}_{\mu }+{\hat{K}}_{\mu }{\hat{U}}_{\mu }\right),$$

where

(A2) $$I=\left[\begin{array}{c} \;\;\;\vdots \\ 1\;0\\ 0\;1\\ \;\;\;\vdots \end{array}\right],$$

${\hat{M}}_{\mu }$ and ${\hat{K}}_{\mu }$ denote the mass matrix and stiffness matrix, respectively. ${\hat{U}}_{\mu }$ stands for the vector of nodal values obtained after imposing the periodic boundary condition.

In this paper, rotational rigid body modes are not taken into consideration. Given that I represents a rigid body mode, one can find that

(A3) $$I^{T}{\hat{K}}_{\mu }^{T}={\left({\hat{K}}_{\mu }I\right)}^{T}=0.$$

Considering the rigid body motion, the displacements can be described as

(A4) $${\hat{U}}_{\mu }=Ic+{\hat{U}}_{\mu }^{*},$$

where ${\hat{U}}_{\mu }^{*}$ represents the node value vector of the subsystem after imposing the restrictions and c denotes a rigid body translation.

By multiplying $N_{\mu }$ on both sides of Equation (A4), one can obtain

(A5) $$N_{\mu }Ic=N_{\mu }{\hat{U}}_{\mu }-N_{\mu }{\hat{U}}_{\mu }^{*},$$

where

(A6) $$N_{\mu }I=I,$$

(A7) $$N_{\mu }{\hat{U}}_{\mu }=u,$$

where $N_{\mu }$ is the shape function. I is a unit matrix, and it should be noted that its size is determined by the equation. Since the design domain consists of specified nodes and retained nodes, we have

(A8) $$I=\left[\begin{array}{c} I_{1}\\ I_{2} \end{array}\right],$$

(A9) $${\hat{U}}_{\mu }=\left[\begin{array}{c} {\hat{U}}_{\mu 1}\\ {\hat{U}}_{\mu 2} \end{array}\right],$$

(A10) $$N_{\mu }=\left[\begin{array}{c} N_{\mu 1}\;N_{\mu 2} \end{array}\right].$$

Then,

(A11) $${\hat{U}}_{\mu 1}=\;I_{1}c+{\hat{U}}_{\mu 1}^{*},$$

(A12) $${\hat{U}}_{\mu 2}=\;I_{2}c+{\hat{U}}_{\mu 2}^{*}.$$

Through Equations (A5) to (A12), one can obtain

(A13) $$c=u-N_{\mu 1}{\hat{U}}_{\mu 1}^{*}-N_{\mu 2}{\hat{U}}_{\mu 2}^{*}.$$

Since ${{\hat{K}}_{\mu }}^{T}={\hat{K}}_{\mu }$, substituting Equations (A3) and (A9) into (A1), one finds that

(A14) $$\begin{array}{c} \beta =I^{T}\left[\begin{array}{c} {\hat{M}}_{11}\;{\hat{M}}_{12}\\ {\hat{M}}_{21}\;{\hat{M}}_{22} \end{array}\right]\left[\begin{array}{c} {\ddot{\hat{U}}}_{\mu 1}\\ {\ddot{\hat{U}}}_{\mu 2} \end{array}\right]. \end{array}$$

From the second equation in system (7), using $\hat{\left(\cdot \right)}$ to indicate that periodic boundary conditions are imposed, one can obtain

(A15) $${\hat{M}}_{22}{\ddot{\hat{U}}}_{\mu 2}+{\hat{K}}_{22}{\hat{U}}_{\mu 2}=-\left({\hat{M}}_{21}{\ddot{\hat{U}}}_{\mu 1}+{\hat{K}}_{21}{\hat{U}}_{\mu 1}\right).$$

Substituting Equation (A12) into the left side of Equation (A15) yields

(A16) $${\hat{M}}_{22}{\ddot{\hat{U}}}_{\mu 2}+{\hat{K}}_{22}{\hat{U}}_{\mu 2}={\hat{M}}_{22}\left(I_{2}\ddot{c}+{\ddot{\hat{U}}}_{\mu 2}^{*}\right)+{\hat{K}}_{22}\left(I_{2}c+{\hat{U}}_{\mu 2}^{*}\right).$$

Substituting Equation (A13) into Equation (A16) yields

(A17) $$\begin{array}{c} \begin{array}{cc} \; & {\hat{M}}_{22}\left(I_{2}\ddot{c}+{\ddot{\hat{U}}}_{\mu 2}^{*}\right)+{\hat{K}}_{22}\left(I_{2}c+{\hat{U}}_{\mu 2}^{*}\right)=\\ & {\hat{M}}_{22}\left(I-I_{2}N_{\mu 2}\right){\ddot{\hat{U}}}_{\mu 2}^{*}+{\hat{K}}_{22}\left(I-I_{2}N_{\mu 2}\right){\ddot{\hat{U}}}_{\mu 2}^{*}\\ & -{\hat{M}}_{22}I_{2}N_{\mu 1}{\ddot{\hat{U}}}_{\mu 1}^{*}-{\hat{K}}_{22}I_{2}N_{\mu 1}{\ddot{\hat{U}}}_{\mu 1}^{*}+{\hat{M}}_{22}I_{2}\ddot{u}+{\hat{K}}_{22}I_{2}u. \end{array} \end{array}$$

In the internal system, it is assumed that the prescribed nodes do not participate in internal vibration; thus,

(A18) $${\hat{U}}_{\mu 1}^{*}=0.$$

Then, the left side of Equation (A15) is

(A19) $$\begin{array}{cc} \; & {\hat{M}}_{22}\left(I-I_{2}N_{\mu 2}\right){\ddot{\hat{U}}}_{\mu 2}^{*}+{\hat{K}}_{22}\left(I-I_{2}N_{\mu 2}\right){\ddot{\hat{U}}}_{\mu 2}^{*}\\ & +{\hat{M}}_{22}I_{2}\ddot{u}+{\hat{K}}_{22}I_{2}u. \end{array}$$

When considering the right side of Equation (A15), setting the prescribed nodes’ displacements as the macroscale displacement u allows one to obtain

(A20) $${\hat{M}}_{21}{\ddot{\hat{U}}}_{\mu 1}+{\hat{K}}_{21}{\hat{U}}_{\mu 1}={\hat{M}}_{21}I_{1}\ddot{u}+{\hat{K}}_{21}I_{1}u,$$

where I is a rigid body mode. Thus,

(A21) $$\begin{array}{c} \left[\begin{array}{c} {\hat{K}}_{11}\;{\hat{K}}_{12}\\ {\hat{K}}_{21}\;{\hat{K}}_{22} \end{array}\right]\left[\begin{array}{c} I_{1}\\ I_{2} \end{array}\right]=0. \end{array}$$

With Equations (A15), (A19), (A20), and (A21), one can derive

(A22) $$\begin{array}{cc} \; & {\hat{M}}_{22}\left(I-I_{2}N_{\mu 2}\right){\ddot{\hat{U}}}_{\mu 2}^{*}+{\hat{K}}_{22}\left(I-I_{2}N_{\mu 2}\right){\hat{U}}_{\mu 2}^{*}=\\ & -\left({\hat{M}}_{21}I_{1}+{\hat{M}}_{22}I_{2}\right)\ddot{u}. \end{array}$$

By multiplying $(I-I_{2}N_{\mu 2}{)}^{T}$ on both sides of Equation (A22), one can obtain

(A23) $$\begin{array}{cc} \; & (I-I_{2}N_{\mu 2}{)}^{T}{\hat{M}}_{22}\left(I-I_{2}N_{\mu 2}\right){\ddot{\hat{U}}}_{\mu 2}^{*}+(I-I_{2}N_{\mu 2}{)}^{T}{\hat{K}}_{22}\\ & \left(I-I_{2}N_{\mu 2}\right){\hat{U}}_{\mu 2}^{*}=-(I-I_{2}N_{\mu 2}{)}^{T}\left({\hat{M}}_{21}I_{1}+{\hat{M}}_{22}I_{2}\right)\ddot{u}. \end{array}$$

Set

(A24) $$M_{\mu 2}^{*}=(I-I_{2}N_{\mu 2}{)}^{T}{\hat{M}}_{22}\left(I-I_{2}N_{\mu 2}\right),$$

(A25) $$K_{\mu 2}^{*}=(I-I_{2}N_{\mu 2}{)}^{T}{\hat{K}}_{22}\left(I-I_{2}N_{\mu 2}\right),$$

(A26) $$D^{T}=(I-I_{2}N_{\mu 2}{)}^{T}\left({\hat{M}}_{21}I_{1}+{\hat{M}}_{22}I_{2}\right),$$

and then Equation (A23) can be simplified as

(A27) $$M_{\mu 2}^{*}{\ddot{\hat{U}}}_{\mu 2}^{*}+K_{\mu 2}^{*}{\hat{U}}_{\mu 2}^{*}=-D^{T}\ddot{u}.$$

Equation (A14) can be described as

(A28) $$\begin{array}{c} \beta =I^{T}\left[\begin{array}{c} {\hat{M}}_{11}{\ddot{\hat{U}}}_{\mu 1}+{\hat{M}}_{12}{\ddot{\hat{U}}}_{\mu 2}\\ {\hat{M}}_{21}{\ddot{\hat{U}}}_{\mu 1}+{\hat{M}}_{22}{\ddot{\hat{U}}}_{\mu 2} \end{array}\right]. \end{array}$$

As mentioned before, the displacements of the prescribed nodes are set as the macroscale displacements u. Together with Equations (A13) and (A18), one can obtain

(A29) $$\begin{array}{cc} \beta = & I^{T}(\left[\begin{array}{c} {\hat{M}}_{11}I_{1}\ddot{u}+{\hat{M}}_{12}I_{2}\ddot{u}\\ {\hat{M}}_{21}I_{1}\ddot{u}+{\hat{M}}_{22}I_{2}\ddot{u} \end{array}\right]\\ & +\left[\begin{array}{c} {\hat{M}}_{12}{\ddot{\hat{U}}}_{\mu 2}^{*}-{\hat{M}}_{12}I_{2}N_{u2}{\ddot{\hat{U}}}_{\mu 2}^{*}\\ {\hat{M}}_{22}{\ddot{\hat{U}}}_{\mu 2}^{*}-{\hat{M}}_{22}I_{2}N_{u2}{\ddot{\hat{U}}}_{\mu 2}^{*} \end{array}\right]), \end{array}$$

where

(A30) $$\begin{array}{c} {\hat{M}}_{\mu }=\left[\begin{array}{c} {\hat{M}}_{11}\;{\hat{M}}_{12}\\ {\hat{M}}_{21}\;{\hat{M}}_{22} \end{array}\right], \end{array}$$

(A31) $$I^{T}=\left[\begin{array}{c} {I_{1}}^{T}\;{I_{2}}^{T} \end{array}\right],$$

yield

(A32) $$\beta =\bar{R}\ddot{u}+D{\ddot{\hat{U}}}_{\mu 2}^{*},$$

where

(A33) $$\bar{R}=I^{T}{\hat{M}}_{\mu }I.$$

## Appendix B. The Map of the Subsystems to the Macroscale

### Appendix B.1. The Map of the Restricted Subsystem to the Macroscale

Consider the generalized eigenvalue problem defined by Equation (14), the solution of which fulfills the mass normalization condition

(A34) $$\left\{\begin{array}{c} \Psi_{\mu 2}^{*T}M_{\mu 2}^{*}\Psi_{\mu 2}^{*}=I\\ \Psi_{\mu 2}^{*T}K_{\mu 2}^{*}\Psi_{\mu 2}^{*}=\Omega_{\mu }^{*2}, \end{array}\right.$$

where

(A35) $$\left\{\begin{array}{c} \Psi_{\mu 2}^{*}=\left[{\hat{\psi }}_{\mu 2}^{*(1)},{\hat{\psi }}_{\mu 2}^{*(2)},\cdots ,{\hat{\psi }}_{\mu 2}^{*(N)}\right]\\ \Omega_{\mu }^{*}=\mathrm{diag}\left[\omega_{\mu }^{*(1)},\omega_{\mu }^{*(2)},\cdots ,\omega_{\mu }^{*(N)}\right], \end{array}\right.$$

where ${\hat{\psi }}_{\mu 2}^{*(N)}$ denotes the *N*th natural vibration mode and $\omega_{\mu }^{*(N)}$ represents the corresponding eigenfrequency.

Using $q_{\mu 2}^{*}$ to denote the amplitude of the eigenmodes, the solution ${\hat{U}}_{\mu 2}^{*}$ can be obtained as

(A36) $${\hat{U}}_{\mu 2}^{*}=\Psi_{\mu 2}^{*}q_{\mu 2}^{*}.$$

Substituting Equation (A36) into Equation (11) and then multiplying both sides of Equation (11) by $\Psi_{\mu 2}^{*T}$, one can obtain

(A37) $$\Psi_{\mu 2}^{*T}M_{\mu 2}^{*}\Psi_{\mu 2}^{*}{\ddot{q}}_{\mu 2}^{*}+\Psi_{\mu 2}^{*T}K_{\mu 2}^{*}\Psi_{\mu 2}^{*}q_{\mu 2}^{*}=-\Psi_{\mu 2}^{*T}D^{T}\ddot{u}.$$

Set

(A38) $$Q=D\Psi_{\mu 2}^{*},$$

and by taking Equation (A34) into consideration, one can obtain

(A39) $${\ddot{q}}_{\mu 2}^{*}+\Omega_{\mu }^{*2}q_{\mu 2}^{*}=-Q^{T}\ddot{u},$$

where $q_{u2}^{*}$ represents the modal amplitude vector. ω is used to denote the excitation macroscale frequency, so

(A40) $${\ddot{q}}_{\mu 2}^{*}=-\omega^{2}q_{\mu 2}^{*}.$$

Through Equations (A38) and (A39), one can derive

(A41) $$q_{\mu 2}^{*}={\left(\omega^{2}I-\Omega_{\mu }^{*2}\right)}^{-1}Q^{T}\ddot{u}.$$

Substituting Equation (A36) into Equation (12) and taking Equation (A40) into consideration, the macroscale inertial force is

(A42) $$\beta =\bar{R}\ddot{u}+Qq_{\mu 2}^{*}.$$

Through Equations (A41) and (A42), one can obtain

(A43) $$\beta =\bar{R}\ddot{u}+Q{\left(\Omega_{\mu }^{*2}/\omega^{2}-I\right)}^{-1}Q^{T}\ddot{u},$$

where $\bar{R}$ represents the effective density tensor defined in Appendix A. Using $R^{e}$ as an effective, frequency-dependent pseudo-density tensor, it can be given as

(A44) $$R^{e}=\bar{R}+\tilde{R},$$

where

(A45) $$\tilde{R}=Q{\left(\Omega_{\mu }^{*2}/\omega^{2}-I\right)}^{-1}Q^{T}.$$

### Appendix B.2. The Map of the Unrestricted Subsystem to the Macroscale

Consider the generalized eigenvalue problem formed by the unrestricted subsystem. Similar to the restricted subsystem, through the natural frequencies and the mass-normalized eigenmodes, the following equation can be obtained in the unrestricted subsystem:

(A46) $$\Psi_{\mu }^{T}{\hat{M}}_{\mu }\Psi_{\mu }{\ddot{q}}_{\mu }+\Psi_{\mu }^{T}{\hat{K}}_{\mu }\Psi_{\mu }q_{\mu }-\Psi_{\mu }^{T}N_{\mu }^{T}\beta =0,$$

yielding

(A47) $${\ddot{q}}_{\mu }+\Omega_{\mu }^{2}q_{\mu }-\Psi_{\mu }^{T}N_{\mu }^{T}\beta =0,$$

where

(A48) $$\left\{\begin{array}{c} \Psi_{\mu }=\left[{\hat{\psi }}_{\mu }^{\left(1\right)},{\hat{\psi }}_{\mu }^{\left(2\right)},\cdots ,{\hat{\psi }}_{\mu }^{\left(N\right)}\right]\\ \Omega_{\mu }=\mathrm{diag}\left[\omega_{\mu }^{\left(1\right)},\omega_{\mu }^{\left(2\right)},\cdots ,\omega_{\mu }^{\left(N\right)}\right], \end{array}\right.$$

where ${\ddot{q}}_{\mu }$ is the modal amplitude vector of the unrestricted subsystem

(A49) $${\ddot{q}}_{\mu }=-\omega^{2}q_{\mu }.$$

Substituting Equation (A49) into Equation (A47) and multiplying both sides of Equation (A47) by $\Psi_{\mu }$, one can obtain

(A50) $$\Psi_{\mu }q_{\mu }=\frac{\Psi_{\mu }{\Psi_{\mu }}^{T}}{\Omega_{\mu }^{2}-\omega^{2}}N_{\mu }^{T}\beta ,$$

with

(A51) $$\left\{\begin{array}{c} {\hat{U}}_{\mu }=\Psi q_{\mu }\\ {\hat{U}}_{\mu }=Ue^{i(\kappa n_{\kappa }\cdot x-\omega t)}. \end{array}\right.$$

Through Equations (A50) and (A51), one can obtain

(A52) $$\Psi_{\mu }{\Psi_{\mu }}^{T}N_{\mu }^{T}\beta =(\Omega_{\mu }^{2}-\omega^{2})Ue^{i(\kappa n_{\kappa }\cdot x-\omega t)}.$$

## Appendix C. Detailed Derivation of the Sensitivity of the Stiffness and Mass Matrices

The global stiffness matrix or global mass matrix can be assembled by the element stiffness matrix or element mass matrix, respectively. The design domain contains *p* kinds of materials, described as

(A53) $$\chi =\left[\chi_{1},\chi_{2},\cdots ,\chi_{p}\right].$$

One can compute the element stiffness matrix $K_{e}$ by

(A54) $$K_{e}=\chi_{1}K_{e}^{1}+\chi_{2}K_{e}^{2}+\cdots +\chi_{p}K_{e}^{p},$$

where $K_{e}^{m},m=1,2,\cdots ,p$ represents the *m*th material’s contribution to $K_{e}$.

Similarly, the element mass matrix $M_{e}$ can be calculated by

(A55) $$M_{e}=\chi_{1}M_{e}^{1}+\chi_{2}M_{e}^{2}+\cdots +\chi_{p}M_{e}^{p},$$

where $M_{e}^{m},m=1,2,\cdots ,p$ represents the *m*th material’s contribution to $M_{e}$.

Using p=3 as an example, three materials are represented by two level-set functions. Using Equation (24), $K_{e}$ and $M_{e}$ can be represented as

(A56) $$\begin{array}{cc} \; & K_{e}=H\left(\phi_{1}\right)\left(1-H\left(\phi_{2}\right)\right)K_{e}^{1}+H\left(\phi_{2}\right)\left(1-H\left(\phi_{1}\right)\right)K_{e}^{2}+\\ & \left(1-H\left(\phi_{1}\right)\left(1-H\left(\phi_{2}\right)\right)-H\left(\phi_{2}\right)\left(1-H\left(\phi_{1}\right)\right)\right)K_{e}^{3}, \end{array}$$

and

(A57) $$\begin{array}{cc} \; & M_{e}=H\left(\phi_{1}\right)\left(1-H\left(\phi_{2}\right)\right)M_{e}^{1}+H\left(\phi_{2}\right)\left(1-H\left(\phi_{1}\right)\right)M_{e}^{2}+\\ & \left(1-H\left(\phi_{1}\right)\left(1-H\left(\phi_{2}\right)\right)-H\left(\phi_{2}\right)\left(1-H\left(\phi_{1}\right)\right)\right)M_{e}^{3}. \end{array}$$

Using $H_{m}$ to denote $H(\phi_{m})$, the sensitivity of $K_{e}$ with respect to $\phi_{1}$ can be calculated as

(A58) $$\frac{\partial K_{e}}{\partial \phi_{1}}=\left(\left(1-H_{2}\right)K_{e1}-H_{2}K_{e2}+\left(2H_{2}-1\right)K_{e3}\right)\delta_{1}.$$

The sensitivity of $K_{e}$ with respect to $\phi_{2}$ is

(A59) $$\frac{\partial K_{e}}{\partial \phi_{2}}=\left(\left(1-H_{1}\right)K_{e1}-H_{1}K_{e2}+\left(2H_{1}-1\right)K_{e3}\right)\delta_{2}.$$

Similarly, the sensitivities of $M_{e}$ with respect to $\phi_{1}$ and $\phi_{2}$ are

(A60) $$\frac{\partial M_{e}}{\partial \phi_{1}}=\left(\left(1-H_{2}\right)M_{e1}-H_{2}M_{e2}+\left(2H_{2}-1\right)M_{e3}\right)\delta_{1},$$

and

(A61) $$\frac{\partial M_{e}}{\partial \phi_{2}}=\left(\left(1-H_{1}\right)M_{e1}-H_{1}M_{e2}+\left(2H_{1}-1\right)M_{e3}\right)\delta_{2},$$

respectively.

In Equations (A58) to (A61), $\delta_{i},i=1,2$ is the derivative of $H(\phi_{i}),i=1,2$, defined in Equation (36).

Using Equation (27), the sensitivity of $K_{e}$ with respect to $\alpha_{m}$ is

(A62) $$\frac{\partial K_{e}}{\partial \alpha_{m}}=g{\left(x\right)}^{-1}\frac{\partial K_{e}}{\partial \phi_{m}}.$$

The sensitivity of $M_{e}$ with respect to $\alpha_{m}$ is

(A63) $$\frac{\partial M_{e}}{\partial \alpha_{m}}=g{\left(x\right)}^{-1}\frac{\partial M_{e}}{\partial \phi_{m}}.$$
